# Editorial: Mitochondrial bioenergetics and metabolism: implication for human health and disease

**DOI:** 10.3389/fmolb.2024.1468758

**Published:** 2024-08-01

**Authors:** Sofia Ahola

**Affiliations:** Institute for Metabolism and Systems Research, University of Birmingham, Birmingham, United Kingdom

**Keywords:** mitochondria, mitochondrial dysfunction, integrated stress response, mitochondrial-ER communication, calcium regulation, inflammation, cell death

Mitochondria are the central metabolic hubs in eucaryotic cells playing a central role in fundamental energy transformation processes such as tricarboxylic acid (TCA) cycle, oxidative phosphorylation (OXPHOS) and iron-sulphur cluster biogenesis. Mitochondria can dictate cellular differentiation and growth by coordinating the cellular metabolic status by, for example, modulating the ATP production rate, producing macromolecules for the cell and serving as an ion buffering compartment. Mitochondria have the power to drive phenotypic change in cells, but also act as the triggers for cell death. Several quality control mechanisms ensure the proper mitochondrial function and are designed to monitor and repair mitochondrial defects. Mitochondrial chaperones and proteases alleviate proteotoxic stress and activate transcriptional defence programs such as Integrated Stress Response (ISR). Fission and fusion of the mitochondrial network allows compartmentalisation of defects and fragmented mitochondria can be taken up by the autophagosomes. Despite all the protective mechanism, mitochondrial dysfunction can persist and eventually trigger cell death. Primary mitochondrial diseases that directly or indirectly affect OXPHOS function are complex multisystem disorders with exceptional clinical variety and involvement of different combinations of affected tissues. Mitochondrial dysfunction is also found as one of the underlaying causes in several other human diseases such as diabetes, cancer, different neurodegenerative diseases and is even playing a part in normal ageing.

This Research Topic emphasizes the intricate role of mitochondria in overall human health and disease. It is essential to gain a deeper understanding of the multifaceted regulation of mitochondria and their connections with other organelles to define their role in common diseases. When studying mitochondria, it is crucial to consider them within the context of the entire cell and understand their interactions with other organelles. “No man is an island, entire of itself” holds true for mitochondria as well. Mitochondria continuously receive signals and metabolites from other cell organelles to fine-tune their function and align with the needs of the cell. Intriguingly, highly similar mechanisms seem to be driving the stress responses in secondary mitochondrial dysfunctions, as has been shown to be essential in primary mitochondrial disorders. Several kinase pathways, such as the AMPK pathway, ISR, and Ca^2+^ -mediated signalling, as well as the induction of inflammation, have been shown to be involved. This suggests that mitochondrial dysfunction-induced cellular metabolic crises are not solely caused by the loss of OXPHOS but also by the widespread effects on cell signalling.

In this Research Topic Ronayne and Latorre-Muro elegantly describe the mitochondria-ER communication and its implications to human health. The endoplasmic reticulum (ER) and mitochondria communicate through specialised structures known as mitochondrial-associated membranes (MAMs). These contact sites are critical for transferring lipids, Ca^2+^, and other signalling molecules, facilitating metabolic coordination and cellular homeostasis. MAMs play a crucial role in mitochondrial dynamics, and the regulation of apoptotic pathways but they are also the sight for coordination of dual translation machineries. Nuclear transcripts for mitochondrial proteins as well as mitochondrial DNA derived transcripts need to be translated in a regulated manner to avoid proteotoxicity. Being the site of protein homeostasis regulation, MAMs are a critical site for inducing ISR and UPR. In this review Ronayne and Latorre-Muro also link a defective ISR induction to age associated diseases such as obesity, diabetes and cancer.

MAMs are also a site for Ca^2+^ and lipid exchange. Mitochondria act as buffers for cellular Ca^2+^, taking up and releasing Ca^2+^ to modulate intracellular signalling pathways. This process is tightly regulated by several transporters and channels, including the mitochondrial calcium uniporter (MCU), which facilitates Ca^2^⁺ entry into the mitochondrial matrix. Elevated mitochondrial Ca^2+^ levels enhance the activity of key dehydrogenases in the TCA cycle, thus boosting ATP production. Dysregulated Ca^2+^ homeostasis can lead to mitochondrial dysfunction and production of reactive oxygen species (ROS) contributing to the pathogenesis of various diseases. For instance, excessive mitochondrial Ca^2^⁺ uptake can trigger the opening of the mitochondrial permeability transition pore (mPTP), leading to cell death and is implicated in conditions such as neurodegeneration, cardiac ischemia-reperfusion injury, and muscular dystrophies. D’Angelo et al. provide a thorough review of the current knowledge on Ca^2^⁺ regulation and MCU shedding the light on the role of Ca^2+^ in cell death mechanisms and ROS production.

Mitochondrial Ca^2+^ homeostasis, MAMs, apoptosis, morphology, and ROS signalling are all targets of kinase-mediated regulation. Phosphorylation by kinases is a crucial mechanism that modifies protein function and influences pathway responses in metabolic processes. By affecting energy production and mitochondrial function, these pathways play a pivotal role in enabling cancer cells to meet their high metabolic demands and evade cell death. Skalka et al. offer an excellent review that explores the main kinase pathways governing mitochondrial function and highlights the current understanding of how mitochondrial metabolism influences cancer cell growth.

Mitochondrial metabolic adaptations are especially crucial in the cell types that are either heavily relying on oxidative metabolism such as cardiomyocytes and β-cells or need the regulation from glycolytic to oxidative metabolism (or *vice versa*) to differentiate as in osteoblasts, myoblasts and immune cells. In their review, Wang et al. dive into the Research Topic of ATP induced cell death in osteoporosis supporting the hypothesis of extracellular ATP increase driving several programmed cell death pathways. Extracellular ATP can trigger cell death, for example, via P2 or Ca^2+^ receptors, via inflammatory pathways or dissipating the mitochondrial membrane potential. They also highlight how in prolonged hyperglycaemia in diabetes contributes to reduced bone density and osteoporosis by impairing the osteoblast and osteoclast function. Diabetes and prolonged stress in glucose handling, is also the main focus of a review by Rivera Nieves et al. In their comprehensive review, the authors discuss about the role of mitochondrial bioenergetics and the special metabolic needs of a β-cell in producing insulin and how mitochondrial dysfunction can contribute to diabetes. A combination of stressors such as gluco- and lipotoxicity, proteostatic stress, Ca^2+^ dysregulation and inflammation can lead to a weakened cell viability and eventually cell death of a β-cell. Interestingly, normal cellular levels of ROS regulate glucose stimulated insulin release but increased levels of ROS upon mitochondrial dysfunction can lead to cell death.

Autoimmunity and cytotoxic T cells mediate β-cell death in diabetes, but also mitochondrial DNA (mtDNA) released into the cytosol can trigger inflammatory responses. Mitochondrial dysfunction, marked by impaired bioenergetics, excessive ROS production, and mtDNA release, is implicated in chronic inflammatory diseases. Similarly, mitochondrial dysfunction in adipocytes and macrophages is associated with the low-grade inflammation observed in obesity and metabolic syndrome. Todosenko et al. offer an intriguing perspective by examining the inflammatory response and the interaction between macrophages and adipocytes in obesity. They highlight the role of Prohibitins in modulating the intercellular cascades of inflammatory responses and analyse the contributions of these two cell types, which are major drivers of inflammation in the pathogenesis of obesity.

Mitochondrial bioenergetics and metabolism are fundamental to cellular function and organismal health. The intricate regulation of mitochondrial activities, particularly Ca^2+^ homeostasis, MAMs, and the roles of mitochondria in inflammation and cell death, underscores their importance in health and disease. Disruptions in these processes can lead to a wide array of pathologies, from metabolic disorders to chronic inflammatory diseases. Understanding these complex mitochondrial functions offers potential therapeutic avenues for treating mitochondrial-related diseases and improving human health.

